# Antioxidant and Antimicrobial Activities of Ethanol Extract from the Stem and Leaf of *Impatiens balsamina* L*.* (Balsaminaceae) at Different Harvest Times

**DOI:** 10.3390/molecules18066356

**Published:** 2013-05-29

**Authors:** Suk-Nam Kang, Young-Min Goo, Mi-Ra Yang, Rashid Ismael Hag Ibrahim, Jae-Hyeon Cho, Il-Suk Kim, Ok-Hwan Lee

**Affiliations:** 1 Department of Animal Resources Technology, Gyeongnam National University of Science and Technology, Gyeongnam 660-758, Korea; E-Mails: whitenightt@hanmail.net (S.-N.K.); karisto2000@nate.com (M.-R.Y.); 2 Sanchung Oriental Medicinal Herb Institute, Chancheong 666-831, Korea; E-Mail: dudals1109@naver.com; 3 Department of Horticulture, Gyeongnam National University of Science and Technology, Gyeongnam 660-758, Korea; E-Mail: rihagibrahim@gmail.com; 4 Institute of Life Science, College of Veterinary Medicine, Gyeongsang National University, Jinju 660-701, Korea; E-Mail: jaehcho@gsnu.ac.kr; 5 Department of Food Science and Biotechnology, Kangwon National University, Chuncheon 200-701, Korea

**Keywords:** *Impatiens balsamina*, phenolic, flavonoid, antioxidant activity, antimicrobial activity

## Abstract

The aim of this study was to investigate the total phenolic content, total flavonoid contents, antioxidant activity and antimicrobial activity of ethanolic extract from stems (S) and leaves (L) of *Impatiens balsamina* L. (Balsaminaceae), which were harvested in Korea on March 10, 2011 (S1 and L1), May 14, 2011 (S2 and L2), and July 5, 2011 (S3 and L3), respectively. Our results revealed that the total phenolic (79.55–103.94 mg CE/g extract) and flavonoid (57.43–104.28 mg QE/g extract) contents of leaf extract were higher (*p* < 0.01) than those of stem extract. Leaf extracts (L1, L2, and L3) exhibited stronger (*p* < 0.01) free radical scavenging activity (66.06, 63.71, and 72.19%, respectively) than that of the positive control. In terms of antimicrobial activity, leaf extracts showed higher inhibitory effects against microorganisms than those of stem extracts (S1, S2, and S3). Among the leaf extracts at different harvest times, L3 showed the greatest antimicrobial activity against both Gram negative and Gram positive strains. From these results, the leaf extract from *I. balsamina* L. might be a valuable bioactive resource, and would seem to be applicable as a natural antioxidant in food preservation.

## 1. Introduction

The addition of synthetic antioxidants such as butylated hydroxyanisole (BHA), butylated hydroxytoluene (BHT) and tertiary butylhydroquinone (TBHQ) to delay lipid oxidation in food is reported to be very effective [1]. However, the utilization of these synthetic antioxidants has been restricted, because of possible health risks and toxicity [2]; additionally, consumers are increasingly demanding additive-free or natural products [3].

*Impatiens balsamina* L. (Balsaminaceae) is an annual herb grown as an ornamental garden plant. The plant is traditionally used to treat thorn or glass-puncture wounds, abscesses [4], scrofulosis, carbuncles, dysentery [5], rheumatism, isthmus and crural aches, fractures, superficial infections, fingernail inflammation [6], tumor, difficult labor and puerperal pain [7]. 

For centuries, preparations from the aerial parts of *I. balsamina* L. have been used in traditional Chinese medicine for antimicrobial, antirheumatic, antipruritic, anti-inflammatory, anti-allergic activities and antitumoral purposes, as well as for the treatment of difficult labor and puerperal pain [8,9,10]. Many compounds have been isolated from *I. balsamina* L., including phenolics [11], flavonols [12], anthocyanin pigments [13], and saponins [14]. While the antipruritic and antianaphylactic properties of some compounds (particularly phenolics and quinones) from this plant have been studied extensively [15], the phytochemical bases of other traditional uses of *I. balsamina* L. extracts have received little attention [5]. Traditionally, the dried herb is either boiled in water to make tea to treat systemic bacterial and fungal infections or applied directly on the skin or nails in a plaster form to treat local infections [16,17].

In this paper, we present the results of antioxidant and antimicrobial characterization of the ethanol extract from *I. balsamina* L. that were cultivated in Sanchung (Gyoungnam, Korea) and harvested at different periods from March 2011 to July 2011. The purpose of this study was to understand how delayed harvest influences the antioxidant and antimicrobial characteristics of ethanol extracts from the steam and leaf of *I. balsamina* L. in a subtropical region.

## 2. Results and Discussion

### 2.1. Total Phenolic and Flavonoid Content

The vacuoles in plant cells constitute the main compartment in which phenolic compounds accumulate [18]. The analysis of total phenolic and flavonoid contents in the ethanol extract from *I. balsamina* L. stem and leaf at different harvest time is shown in Table 1. The total phenolic contents of the extracts were shown as (+)-catechin equivalents and flavonoids as quercetin equivalents. The total phenolic contents of S1, S2, and S3 were 18.95, 17.02, and 12.92 mg CE/g dry weight, and in L1, L2, and L3 were 79.55, 80.76, and 103.94 mg CE/g dry weight, respectively. The flavonoid contents of S1, S2, and S3 were 6.38, 8.13, and 4.28 mg QE/g dry weight, and in L1, L2, and L3 were 57.43, 67.22, and 104.28 mg QE/g dry weight, respectively. These results revealed that, as a whole, the total phenolic and flavonoid contents of leaf extracts were higher (*p* < 0.01) than those of stem extracts. Both phenolic and flavonoid compounds from *I. balsamina* L. are known to have diverse biological activities and may also be responsible for the radical-linked antioxidant effects and of *I. balsamina* L. [10]. Therefore, these results indicate that high flavonoids and phenolic compounds in leaf extracts may account for their strong antioxidant and antimicrobial activities compared with stem extracts. As of harvest time, the phenolic contents of the later harvested stems (S3) were lower than the earlier harvested stems (S1 and S2), whereas the later harvested leaves (L3) contained higher total phenolic and flavonoid contents than earlier harvested leaves (L1 and L2). These results showed that the phenolic contents of stems were significantly decreased (*p* < 0.01) with delayed harvest time, but at the same time, the total phenolic and flavonoid contents of leaves were significantly increased (*p* < 0.01) when the harvest time was delayed.

**Table 1:** The total phenolic content (TPC) and flavonoid content of 70% ethanol extracts from *I. balsamina* L. at different harvest time.

Samples ^1)^	TPC (mg CE/g extract)	Flavonoid (mg QE/g extract)
S1	18.95 ± 3.08 ^c^	6.38 ± 1.60 ^e^
S2	17.02 ± 3.31 ^c^	8.13 ± 1.62 ^d^
S3	12.92 ± 3.25 ^d^	4.28 ± 2.16 ^f^
L1	79.55 ± 4.75 ^b^	57.43 ± 2.58 ^c^
L2	80.76 ± 5.32 ^b^	67.22 ± 2.16 ^b^
L3	103.94 ± 5.83 ^a^	104.28 ± 3.60 ^a^

Note: ^1)^ S1 and L1 are *Impatiens balsamina* L. stem and leaf, respectively, harvested on March 10, 2011. S2 and L2 are *Impatiens balsamina* L. stem and leaf, respectively, harvested on May 14, 2011. S3 and L3 are *Impatiens balsamina* L. stem and leaf, respectively, harvested on July 5, 2011. TPC means total phenolic content. ^a–e^ Means ± SD deviation were significantly different within the same column (*p* < 0.01).

### 2.2. Free Radical Scavenging Activity

DPPH is a stable free radical, and researchers have been using its quenching reaction to evaluate the efficiency of antioxidants [19,20,21]. The DPPH radical has been widely used to test the free radical scavenging ability (FRSA) of various natural products and has been accepted as a model compound for free lipids-originating radicals [22]. The inhibition of DPPH radicals was determined by the antioxidant-induced decrease in their absorbance at 515 nm. The actual reaction taking place between the DPPH stable radical and the antioxidant (AH) is DPPH• + (AH)_n_ → DPPH-H + (A•)_n_. Although free radicals formed (A•) in general are less reactive, a stable molecule can be generated by radical–radical interaction, depending on the structure of the molecule.

**Figure 1:**
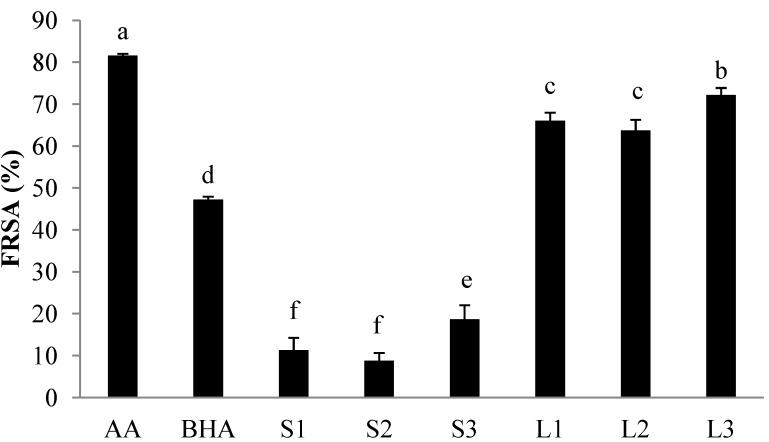
DPPH radical scavenging activity (FRSA) of ethanol extracts (0.1 mg/mL) of *I. balsamina* L. at different harvest times. ^a–f^ Values are the means ± SD of 3 samples. Bars with different letters indicate statistically significant difference among groups at *p* < 0.01. S1, S2, and S3 are stems and L1, L2 and L3 are leaves of *Impatiens balsamina* L. harvested on March 10, 2011, May 14, 2011 and July 5, 2011, respectively. AA: ascorbic acid, BHA: butylated hydroxyanisole.

The analysis of FRSAs in the ethanol extract from *I. balsamina* L. is shown in Figure 1. In this experiment, BHA and ascorbic acid were used as references (positive control). In the DPPH test, the FRSAs of BHA and ascorbic acid were 28.35% and 79.39%, respectively, at a concentration of 0.1 mg/mL. The plant, in general, showed high antioxidant capacities and significant differences (*p* < 0.01) between different samples were observed at the same concentration (0.1 mg/mL). These results clearly indicate that all tested samples exhibited antioxidant activity as follows from higher to lower (*p* < 0.01): Ascorbic acid > L3 (54.05%) > L1 and L2 (44.84–45.14%) > BHA > S1, S2 and S3 (6.64–9.37%). As a whole, the FRSAs of leaf extracts were higher than those of stem extracts at the same concentration. 

The FRSA increased significantly (*p* < 0.01) to 5.2-fold, from a mean of 12.93 mg/mL in stems to a mean of 67.32 mg/mL in leaves. In addition, these result indicated that FRASs of stem and leaf extracts were significantly increased (*p* < 0.01) as time of harvest was delayed. Phenolic antioxidants have been recognized to function as electron or hydrogen donors [23], thus, the free radical scavenging activity of these phenolic compounds might be mostly related to their phenolic hydroxyl groups. From the results of this study, the total phenolic contents and free radical scavenging activity were very closely correlated for all sets of samples (*p* < 0.01, r = 0.979), and flavonoid contents and free radical scavenging activity were also very closely correlated (*p* < 0.01, r = 0.944). 

### 2.3. Hydroxyl Radical Scavenging Activity

**Figure 2:**
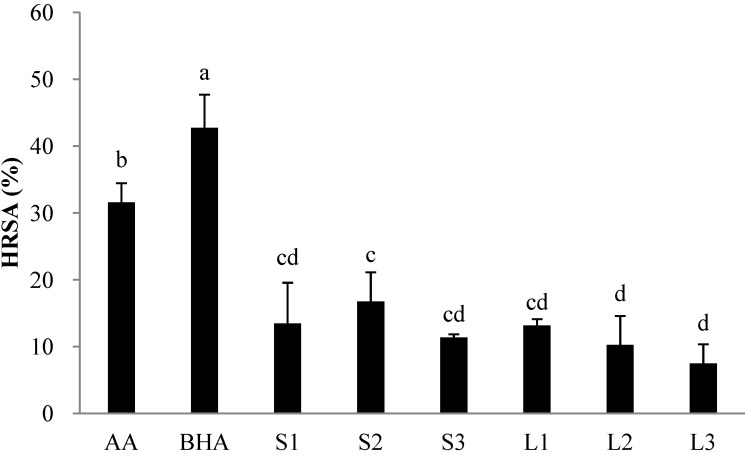
Hydroxyl radical scavenging activities (HRSA) of ethanol extracts (0.1 μg/mL) of *I. balsamina* L. harvested at different time. S1, S2, and S3 are stems and L1, L2 and L3 are leaves of *I. balsamina* L. harvested on March 10, 2011, May 14, 2011 or July 5, 2011, respectively. ^a–d^ Values are the means ± SD of 3 samples. Bars with different letters indicate statistically significant difference among groups at *p* < 0.01.

Among the reactive oxygen species (ROS), hydroxyl radicals are the most reactive and are the predominant radicals generated endogenously during aerobic metabolism to initiate cell damage *in vivo* [24,25]. The inhibitory action of *I. balsamina* L. extracts was examined on deoxyribose degradation, which gives an indication of hydroxyl radical scavenging action [26,27]. The HRSAs of stem and leaf extracts of *I. balsamina* L., harvested at different times, were tested in relation to BHA and ascorbic acid at the same concentration as shown in Figure 2. The HRSA of BHA was 42.74% and of ascorbic acid was 31.59% at the same concentration (0.1 μg/mL). Significantly higher HRSA was observed in the standards (BHA and ascorbic acid) compared to extracts from *I. balsamina* L. stems or leaves at the same concentration. Relatively lower HRSA in extracts of later harvested leaves (L2 and L3) was seen compared to mid-term harvested stem (S2) at the same concentration (1 mg/mL). These results also indicate that hydroxyl radical scavenging activity of stem and leaf extracts from the *I. balsamina* L. showed a different pattern with different methods used such as FRSA. There are many methods for determination of antioxidant capacity and each method has its own limitation. It was shown that some antioxidant assays give different antioxidant activity trends.

### 2.4. Antimicrobial Activity

The antimicrobial activities of *I. balsamina* L. ethanol extract (4 mg/mL) on *S. aureus*, *V. parahaemolyticus*, *B. cereus*, *S. typhimurium*, *L. monocytogenes*, *E. coli*, *C. albicans and C. perfringens* are shown in Table 2. The antimicrobial activities of L1 and L2 extracts had a strong activity against *C. albicans* and *C. perfringens*, clear activity against *V. parahaemolyticus* and *B. cereus*, moderate activity against *S. typhimurium* and *E. coli* and slight activity against *L. monocytogenes*, whereas the antimicrobial activity of L3 extract had a strong activity against *C. albicans* and *C. perfringens*, clear activity against *V. parahaemolyticus*, *B. cereus* and *S. aureus*, and moderate activity against *S*. *typhimurium* , *L. monocytogenes*, and *E. coli*. These results indicated that ethanol leaf extracts from *I. balsamina* L. (L1, L2 and L3) regardless of harvest time possess the greatest inhibitory activity, and they showed strong antimicrobial activity against *C. albicans* and *C. perfringens*, clear antimicrobial activity against *V. parahaemolyticus* and *B. cereus*, and moderate antimicrobial activity against *S. typhimurium* and *E. coli*. These results also confirm that ethanol leaf extracts from *I. balsamina* L. contain a relatively higher antimicrobial activity than the stem extracts at the same concentration (4 mg/mL).

**Table 2:** The antimicrobial activity of 70% ethanol extracts from *I. balsamina* L. (4 mg/mL) at different harvest times.

Microorganism ^1)^	S1	S2	S3	L1	L2	L3
STA	-^2)^	-	-	~	+	++
VIP	-	-	-	++	++	++
BAC	-	-	-	++	++	++
SAT	-	-	-	+	+	+
LIM	+	-	-	~	~	+
ECO	-	-	+++	+	+	+
CAN	+	-	++	+++	+++	+++
CLB	-	-	-	+++	+++	+++

Note: ^1)^ Microorganisms: STA, *Staphylococcus aureus* ATCC12692; VIP, *Vibrio parahaemolyticus* ATCC 17802; BAC, *Bacillus cereus* KFRI 181; SAT, *Salmonella typhimurium* ATCC 14028; LIM, *Listeria monocytogenes* ATCC 1911; ECO, *Escherichia coli* KFRI 836; CAN, *Candida albicans* 00116 KCTC 7007; CLB, *Clostridium perfringens* ATCC 13124. ^2)^ -; No antimicrobial activity, ~; Slight antimicrobial activity inhibition zone (I.Z) of 8–9 mm, +; Moderate antimicrobial activity I.Z of 9.1–12 mm, ++; Clear antimicrobial activity I.Z of 12.1–15 mm, +++; Strong antimicrobial activity I.Z of more than 15 mm.

The stem ethanol extracts of* I. balsamina* L. (S1, S2 and S3) did not show any antimicrobial activity against *S. aureus*, *V. parahaemolyticus*, *B. cereus*, *S. typhimurium*, and *C. perfringens*. However, S1 showed a moderate antimicrobial activity against *L. monocytogenes* and *C. albicans*, and that of S3 expressed a strong antimicrobial activity against *E. coli* and clear antimicrobial activity against *C. albicans*. Tailor *et al.* [28] reported that the antimicrobial and antifungal properties of *I. balsamina* L. were due to cysteine-rich compounds. It is generally considered that the inhibition of microbial growth by an antioxidant may be due to the free radical scavenging activity of phenolic compounds [29,30,31]. From these results, total phenolic content (r = 0.96) and free radical scavenging activity (r = 0.98) were closely correlated to each other (*p* < 0.01). However, there wasn’t any significant correlation between antimicrobial activity and total phenolic contents, flavonoid contents, and free radical scavenging activity among tested samples.

## 3. Experimental

### 3.1. Chemicals and Extracts

Ascorbic acid, potassium persulphate, disodium hydrogen phosphate (Na_2_HPO_4_) 2,2-diphenyl-1-picrylhydrazyl (DPPH), deoxyribose, ferric chloride (FeCl_3_), hypoxanthine, nitroblue tetrazolium (NBT), xanthine oxidase, Folin–Ciocalteu’s phenol reagent, (+)-catechin, quercetin, aluminum chloride (AlCl_3_) were purchased from Sigma Co. (St. Louis, MO, USA). Other solvents used were of analytical grade and purchased from Merck Co. (Darmstadt, Germany). Distilled and deionized water (dd. H_2_O) was prepared by Ultrapure TM water purification system (Lotun Co., Ltd., Taipei, Taiwan). 

### 3.2. Plant Samples

The *I. balsamina* L. was grown in the field on December 10, 2010 at Sanchung (Gyoungnam, Korea). Fresh *I. balsamina* L. stems (S) and leaves (L) were harvested on March 10, 2011 (S1 and L1), May 14, 2011 (S2 and L2), and July 5, 2011 (S3 and L3). Then the stems and leaves were homogenized and freeze-dried.

### 3.3. Extraction of Samples

Dried sample of each plant part (40 g) were extracted with 70% ethanol (400 mL) at room temperature for two weeks. The residues were extracted with 70% ethanol (600 mL), also at room temperature, for three days. Extracts were filtered through Whatman No. 2 filter papers and the two filtratrates were combined and then evaporated at 40 °C to remove the ethanol. The extracts were completely dried in a freeze-drier and stored at −20 °C until further use.

### 3.4. Measurement of Total Phenolic Content Using Folin-Ciocalteu Assay

Total phenolic contents of the extracts were determined spectrophotometrically according to the Folin-Ciocalteu colorimetric method [31].

### 3.5. Measurement of Total Flavonoids

Total flavonoid was determined using the method of Meda *et al.* [32] with minor modifications. In brief, 0.25 mL of sample (0.1 mg/mL) was added to a tube contained 1 mL of double-distilled water followed by 0.075 mL of 5% NaNO_2_, 0.075 mL of 10% AlCl_3_ and 0.5 mL of 1 M NaOH at 0, 5 and 6 min, sequentially. Finally, the volume of the reaction solution was adjusted to 2.5 mL with double-distilled water. The absorbance of the solution was measured at 410 nm wavelength in a spectrophotometer (Ultrospec 2100 pro; Amersham Pharmacia Biotech Co., Piscataway, NJ, USA). Quercetin is a ubiquitous flavonoid, present in many plant extracts, was used as a standard to quantify the total flavonoid content of ethanol extract of the spice and the results were expressed in microgram quercetin equivalents (QE)/gram.

### 3.6. Measurement of Free Radical Scavenging Activity on DPPH Assay

The free radical scavenging activity of samples (1 mg/mL) was measured according to the method of Brand-Williams *et al.* [20]. L-ascorbic acid and BHA were used as a positive control. The inhibition percentage was calculated from the following equation: Inhibition % = [(absorbance of control − absorbance of sample)/absorbance of control] × 100.

### 3.7. Measurement of Hydroxyl Radical Scavenging Activity

The scavenging activity of samples in DMSO on the hydroxyl radical was measured by the deoxyribose method [27] with a slight modification. The deoxyribose assay was performed in 10 mM phosphate buffer (pH 7.4) contained 2.5 mM deoxyribose, 1.5 mM H_2_O_2_, 100 μM FeCl_3_, 104 μM EDTA, and the test sample (10 μg/mL). The reaction was started by adding ascorbic acid to a final concentration of 100 μM and the reaction mixture was incubated for 1 h at 37 °C in a water-bath. The color was developed by the addition of 0.5% thiobarbituric acid followed by ice-cold 2.8% trichloroacetic acid in 25 mM NaOH and heating for 30 min at 80 °C. A control was performed without samples (A1). The sample (A2) was cooled on ice and the absorbance was measured at 532 nm. The hydroxyl radical scavenging activity (HRSA) was calculated by the following equation: HRSA% = (A1 − A2/A1) × 100.

### 3.8. Antimicrobial Activity

The gram-positive bacteria *Bacillus cereus* KFRI 181, *Listeria monocytogenes* ATCC 1911, *Clostridium perfringens* ATCC 13124, and *Staphylococcus aureus* ATCC12692; and the gram negative bacteria *Salmonella typhimurium* ATCC 14028, *Escherichia coli* KFRI 836 and *Vibrio parahaemolyticus* ATCC 17802, and *Candida albicans* 00116 KCTC 7007 were obtained from Korea Food Research Institute (KFRI, Seongnam, Korea) and Korea National Microbiological Research Resources Center (KNMRRC, Suwon, Korea). The samples were dissolved in dimethyl sulfoxide (DMSO) and filtered by 0.45 μm Millipore membrane filters. Antimicrobial test was then carried out by disc diffusion method [17]. Bacterial suspensions of 100 mL each contained 10^8^ CFU/mL were spread on nutrient agar (Difco, Lab., Detroit, MI, USA) medium. The 8-mm diameter discs (Toyo Roshi Kaisha, Ltd., Tokyo, Japan) were impregnated with 200 μL (4 mg/mL) of each samples (dry base) and placed on the inoculated agar. The inoculated plates were incubated at 37 °C for 48 h for clinical bacterial strains. Antimicrobial activity was evaluated by measuring the zone of inhibition against the tested organisms. Each assay in this experiment was repeated three times.

### 3.9. Statistical Analysis

The results were reported as mean ± standard deviation (SD). The significance of differences among means of treatments was determined by analysis of variance (one-way ANOVA) using SAS version 8.1 (SAS Institute, Cary, NC, USA). Correlation analyses were performed using the Pearson’s correlation coefficient^®^.

## 4. Conclusions

In conclusion, we found that the leaf extracts from *I. balsamina* L. had higher total phenolic content, total flavonoids content, DPPH radical scavenging activity and antimicrobial effect than those of stem extracts. The higher antioxidant and antimicrobial activities observed in L3 may be due to the total phenolic and flavonoid contents. Our data suggest that the leaf extract from *I. balsamina* L. are effective in scavenging radicals and protecting microorganisms when assessed by DPPH assay and antimicrobial test. In addition, the total phenolic contents and FRSA of leafs were significantly increased with delayed harvest times. From these results, the leaf extract from *I. balsamina* L. could be utilized to develop natural antioxidant and antimicrobial agent. However, the effect of harvest times on the identification of phenolic compounds in stem and leaf extracts from *I. balsamina* L. is still required. Further studies on these extracts with respect to antioxidant properties *in vivo* are needed.
